# Interactive Effects of Ambient Ozone and Meteorological Factors on Cerebral Infarction: A Five-Year Time-Series Study

**DOI:** 10.3390/toxics13070598

**Published:** 2025-07-16

**Authors:** Yanzhe Chen, Songtai Yang, Hanya Que, Jiamin Liu, Zhe Wang, Na Wang, Yunkun Qin, Meng Li, Fang Zhou

**Affiliations:** 1 Institute of Public Health Surveillance and Evaluation, Zhengzhou Center for Disease Control and Prevention, Zhengzhou 450001, China; zzgongweisuo@163.com (Y.C.); 15003897097@163.com (H.Q.); 2 Department of Occupational Health, College of Public Health, Zhengzhou University, Zhengzhou 450001, China; yangsongtai2022@163.com (S.Y.); liujiamin0417@163.com (J.L.); wangzhe07078@163.com (Z.W.); wfengqiao@zzu.edu.cn (N.W.); 15737361723@163.com (Y.Q.)

**Keywords:** ozone, cerebral infarction, time-series study, interaction, meteorological factors

## Abstract

**Objective:** Our objective was to investigate the short-term effects of ambient ozone (O_3_) meteorological factors and their interactions on hospitalizations for cerebral infarction in Zhengzhou, China. **Methods**: Daily data on air pollutants, meteorological factors, and hospitalization of cerebral infarction patients were collected from 1 January 2019 to 31 December 2023 in Zhengzhou, China. A generalized additive model was constructed to evaluate the association between ambient O_3_ levels and hospitalization for cerebral infarction. A distributed lag non-linear model was applied to capture lagged and non-linear exposure effects. We further examined the modifying roles of temperature, humidity, wind speed, and atmospheric pressure, and conducted stratified analyses by sex, age, and season. **Results**: O_3_ exposure was significantly associated with increased cerebral infarction risk, particularly during the warm season. A bimodal temperature-lag pattern was observed, as follows: moderate temperatures (10–20 °C) were associated with immediate effects, while cold (<10 °C) and hot (>30 °C) temperatures were linked to delayed risks. The association of O_3_ and hospitalizations for cerebral infarction appeared stronger under high humidity, low wind speed, and low atmospheric pressure. **Conclusions**: Short-term O_3_ exposure and adverse meteorological conditions are jointly associated with an elevated risk of cerebral infarction. Integrated air quality and weather-based warning systems are essential for targeted stroke prevention.

## 1. Introduction

Air pollution has emerged as a significant global public health challenge, with extensive evidence linking air pollution exposure to increased rates of illness and mortality [1,2,3,4]. Extended exposure to air pollutants correlates with elevated disease incidence and impaired pulmonary capacity. Fine particulate matter (PM_2.5_), nitrogen dioxide (NO_2_), and sulfur dioxide (SO_2_) can infiltrate deep respiratory tissues and enter systemic circulation, inducing inflammatory responses and aggravating chronic respiratory conditions. Scientific evidence has further established significant associations between air pollutants and the development of cardiovascular disorders [5,6]. Among these pollutants, PM_2.5_ and ozone (O_3_) have become two key indicators for assessing population-level exposure in the global burden of disease studies. In recent years, the concentration of ambient particulate matter (PM) has gradually declined in most Chinese cities, due to effective regulatory efforts, while the levels of ambient O_3_ have shown a consistent upward trend [7]. O_3_ is regarded as a secondary pollutant that primarily arises from industrial emissions, vehicular exhaust, and the release of solvents and chemicals. Numerous studies have demonstrated that short-term exposure to O_3_ is linked to a higher incidence of cardiovascular and cerebrovascular diseases [8,9,10].

Cerebral infarction, commonly known as ischemic stroke, is characterized by the dysfunction of brain cells due to a lack of oxygen and nutrients resulting from inadequate blood supply to the brain. Growing epidemiological evidence has demonstrated a clear link between the incidence of cerebral infarction and various meteorological factors [11,12,13,14,15]. For instance, a study conducted in Japan shows that the incidence of stroke is highest in spring [16]. Similarly, research from Qatar found a significant positive correlation between air temperature and cerebral infarction incidence, whereas a negative correlation was found for relative humidity [17].

Despite these findings, the combined effects and potential interactions between ambient O_3_ and meteorological factors on the risk of cerebral infarction remain insufficiently explored. Most existing studies have focused on the main effects of single environmental exposures, with limited attention given to the modifying or synergistic effects of meteorological variables on air pollution-related health outcomes. Understanding such interactions is essential for clarifying the environmental determinants of cerebrovascular events, especially under the conditions of climate variability and increasing O_3_ levels.

Zhengzhou, located in the northern central part of Henan Province, experiences a warm temperate continental climate and has undergone significant industrialization and urban expansion in recent decades. Although the concentration of SO_2_, NO_2_, and CO in Zhengzhou has shown a gradual decrease and remains lower than the average level of large- and medium-sized cities across China, the concentration of O_3_ has remained persistently high, making O_3_ pollution the primary air quality concern in Zhengzhou City and a significant environmental health risk to its residents.

Given the high incidence of cerebral infarction and persistently elevated ambient O_3_ concentrations in Zhengzhou, this study aimed to evaluate the short-term effects of ambient O_3_ exposure and meteorological conditions on hospitalizations for cerebral infarction, using data from 2019 to 2023. A generalized additive model (GAM) was used to quantify the associations between O_3_ and daily hospital admissions, while a distributed lag non-linear model (DLNM) was used to explore the potential interactions between O_3_ and meteorological variables. Additionally, stratified analyses were conducted to assess potential differences in sex, age group, and season. It is expected that the results of this study will provide a scientific basis for the development of a theoretical framework for cerebral infarction prevention and control, thereby contributing to reducing the burden of cerebral infarction in the future.

## 2. Materials and Methods

### 2.1. Study Population

Hospitalization data were obtained from 4 hospitals located in different districts of Zhengzhou, as follows: Zhengzhou First People’s Hospital, Zhengzhou Second People’s Hospital, Zhengzhou Central Hospital, and Zhengzhou Yi he Hospital. The collected information included admission date, age, sex, and diagnosis codes based on the International Statistical Classification of Diseases and Related Health Problems, 10th Revision (ICD-10). Patients diagnosed with cerebral infarction between 1 January 2019 and 31 December 2023 were identified based on their ICD-10.

### 2.2. Exposure Assessment

Air pollutant data were collected from nine fixed air quality monitoring stations operated by the Zhengzhou Environmental Protection Bureau, with the monitoring instruments and their specifications as follows: O_3_, Thermo Scientific Model 49i ultraviolet photometric O_3_ analyzer (Thermo Fisher Scientific, Waltham, MA, USA); PM_2.5_/PM_10_, Thermo Scientific Model 5030 SHARP synchronous hybrid monitor (Thermo Fisher Scientific, Waltham, MA, USA), SO_2_/NO_2_/CO, Ecotech EC9830 gas analyzer (Ecotech, Knoxfield, VIC, Australia). All instruments undergo mandatory quarterly verification by the Provincial Metrology Institute. Annual drift is kept below 2%, and the data missing rate remains under 1%. Missing values were imputed using spatial interpolation based on adjacent stations.

Meteorological data were sourced from daily reports issued by the Zhengzhou Meteorological Bureau, encompassing metrics such as daily average pressure, temperature, relative humidity, and wind speed. Daily air pollutant concentrations and meteorological data were complete and free of missing values during the study period.

### 2.3. Statistical Analysis

Spearman rank correlation analysis was conducted to examine the relationship between atmospheric pollutants and meteorological factors. Time-series analysis was then used to assess the association between hospital admissions for cerebral infarction and ambient O_3_ levels. The data on atmospheric pollution, meteorological variables, and hospital admissions were all continuous daily variables. The distribution of daily hospitalization counts for cerebral infarction followed a quasi-Poisson distribution, satisfying the requirements for time-series analysis. A GAM with a quasi-Poisson link function was applied to analyze the data. Considering the characteristics of the hospital admission data, we controlled for multiple confounding factors, including long-term trends and meteorological variables. This approach allowed us to quantitatively assess the impact of air pollutants on daily hospital admissions and their lagged effects. Excess risk (*ER*) was calculated to represent the percentage change in daily hospital admissions per 10 µg/m^3^ increase in atmospheric pollutant concentration. The basic model is defined as follows:

Log[*E*(*Yt*)] = *α* + *βZt* + s(time, *df*) + s(temperature, *df*) + s(atmospheric pressure, *df*) + s(relative humidity, *df*) + s(wind speed, *df*) + as.factor (*DOW*)+ as.factor (*Holiday*)

In this model:

*E*(*Yt*) represents the expected value of hospital admissions on day t; *α* is the intercept; *Zt* is the daily average concentration of each atmospheric pollutant on day *t*; the *β* coefficient indicates the relative risk (*RR*) of daily hospital admissions per unit increase in the concentration of each pollutant (*Zt*); *s*() represents a non-parametric smoothing function applied to calendar time, average temperature, average atmospheric pressure, average relative humidity, and average wind speed; *DOW* is included in the model as a dummy variable to account for the weekday effect; and *df* denotes the degrees of freedom. Based on previous studies, we selected 7 *df*/year for calendar time and 3 *df* for the average temperature, average atmospheric pressure, average relative humidity, and average wind speed [18,19].

To further investigate lagged and non-linear effects, we applied a DLNM, which allows for simultaneous modeling of exposure–response and lag–response relationships for O_3_ and meteorological variables. This approach captures potential delayed health effects caused by short-term environmental exposures. *p*-values were corrected for multiple comparisons using the FDR method.

Subgroup analyses were conducted by stratifying the population according to age (≤65 years and >65 years), sex (male and female), and season. Seasons were defined as warm (May to October) and cold (November to April) in accordance with local climate conditions. The z-test was used to evaluate potential differences among these subgroups.

All statistical analyses were conducted using R software (version 4.1.2) with the following packages: “mgcv”, “dlnm”, “splines”, and “ggplot2”. A two-sided *p*-value of less than 0.05 was deemed statistically significant.

## 3. Results

### 3.1. Description Statistics of Daily Hospitalizations for Cerebral Infarction, Air Pollutants, and Meteorological Variables

**Figure 1:**
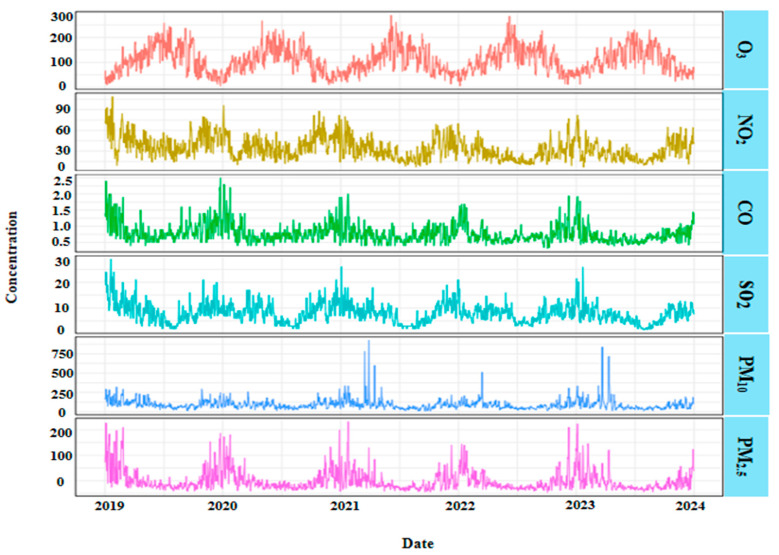
Time-series of the six criteria pollutants concentrations, 2019–2023.

Given the non-normal distribution of hospitalization, air pollutant, and meteorological data, interannual comparisons are presented as median (*P*_25_, *P*_75_) values, with distributional differences assessed using the Kruskal–Wallis test. Key findings from Table 1 include the following: cerebral infarction hospitalizations were significantly higher among males than females; individuals aged 18–65 years accounted for the highest proportion of hospitalizations; hospitalization risk was elevated during warm seasons relative to cold seasons; and, with the exception of O_3_, all pollutants exhibited significant interannual concentration variations (*p* < 0.05), with median values demonstrating a consistent downward trend over the study period. As shown in Figure 1, concentrations of all pollutants except for O_3_ exhibited a declining trend annually, while O_3_ demonstrated distinct seasonal variation, with higher concentrations in warm seasons and lower concentrations in cold seasons.

### 3.2. Correlation Between Air Pollutants and Meteorological Factors

Spearman correlation analysis results are shown in Table 2. Significant positive correlations were found among all pollutants (r = 0.38–0.80, *p* < 0.01), except for O_3_. O_3_ exhibited correlations with SO_2_, NO_2_, CO, PM_10_, and PM_2.5_ (r = −0.38 to −0.18, *p* < 0.01). Furthermore, O_3_ was positively correlated with temperature and wind speed, but negatively correlated with relative humidity and air pressure (*p* < 0.05).

### 3.3. Association Between O_3_ Levels and Daily Cerebral Infarction Hospitalizations

When the lag period was set to 7 days, a significant positive association was found between O_3_ levels and hospitalizations for cerebral infarction. As shown in Table 3 and Table 4, the maximum single-day lag effect was 0.11% (95% CI: 0.08–0.14%), while the maximum cumulative lag effect was 0.13% (95% CI: 0.10–0.16%) for every 10 µg/m^3^ increase in O_3_ concentration.

Stratified analysis based on sex showed that increased O_3_ levels significantly elevated hospitalization risk for both men and women, with the strongest effects observed on lag day 0 and day 01. However, no statistically significant difference was found in effect magnitude between sexes. In terms of age classification, O_3_ exposure was associated with an elevated risk of cerebral infarction across all age groups, with the most pronounced effects in people over 80 years of age. Seasonal analysis shows that the lag effect in the warm season is significantly higher than that in the cold season.

### 3.4. Influence of Meteorological Factors on Cerebral Infarction

**Figure 2:**
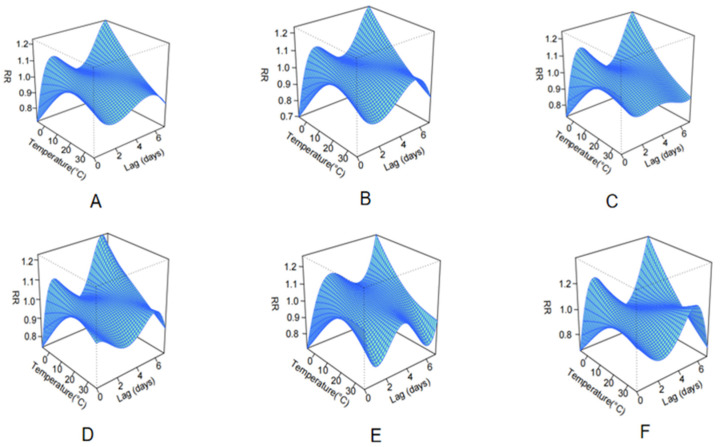
Three-dimensional surface plots of the lagged effects of ambient temperature on cerebral infarction across sex and age subgroups. (**A**) Total; (**B**) Male; (**C**) Female; (**D**) Age: 18–65 years; (**E**) Age: 66–80 years; (**F**) Age: >80 years. Abbreviation: RR, relative risk.

As shown in Figure 2, the analysis revealed a time-varying, non-linear association between ambient temperature and the risk of cerebral infarction. On the day of exposure (lag 0), moderate temperatures (10–20 °C) were associated with the highest relative risk, forming an inverted U-shaped curve. However, at longer lags (lags 2–3), the risk increased under lower temperature conditions, while at lags 4–6, higher temperatures (>30 °C) appeared to be associated with elevated risks, particularly among elderly individuals. These findings suggest a delayed dual-effect of temperature, where both low and high temperatures may influence stroke risk through different lag structures. Stratified analysis showed no substantial difference between males and females, whereas older adults, especially those aged over 80, exhibited greater risk fluctuations over time, reflecting age-specific vulnerability to delayed temperature-related stress.

### 3.5. Interaction Between Meteorological and Pollutant Factors on Cerebral Infarction

**Figure 3:**
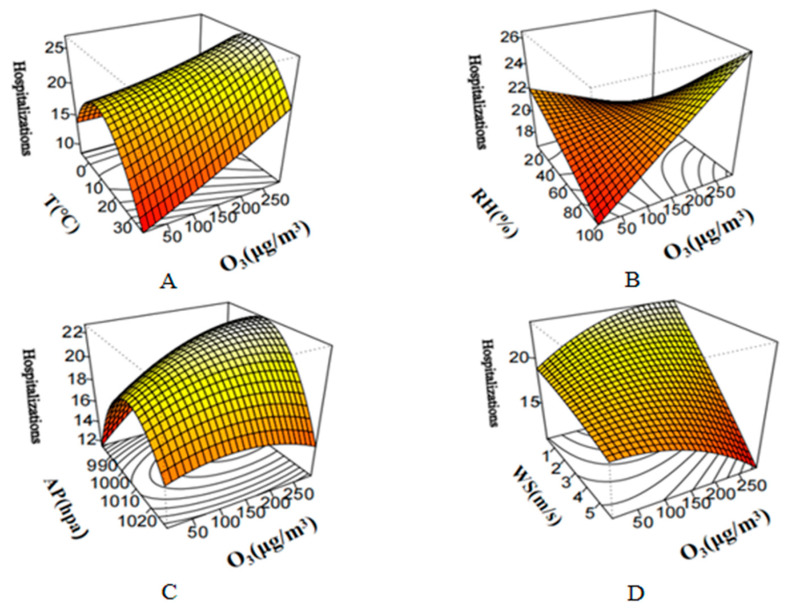
Interaction between meteorological and O_3_ on cerebral infarction. Hospitalizations, hospitalizations for cerebral infarction. (**A**) Temperature–O_3_ Interaction and Cerebral Infarction; (**B**) Relative humidity–O_3_ Interaction and Cerebral Infarction; (**C**) Air pressure–O_3_ Interaction and Cerebral Infarction; (**D**) Wind speed–O_3_ Interaction and Cerebral Infarction. Abbreviations: T, temperature; RH, relative humidity; AP, air pressure; WS, wind speed.

To further study the interaction between O_3_ and meteorological factors, we used the DLNM model to plot a three-dimensional graph. The results are shown in Figure 3. The risk of cerebral infarction increased with rising O_3_ levels under various meteorological conditions. The strongest interaction effect was observed between O_3_ and relative humidity, where hospitalization numbers rose sharply under high humidity (>80%) and high O_3_ conditions. Similarly, low wind speed (<1.5 m/s) and low atmospheric pressure (<1000 hpa) enhanced the effect of O_3_ on hospitalization risk. Notably, the combined effect of moderate temperature (10 °C) and high O_3_ also contributed to increased risk. These findings illustrate a synergistic pattern, whereby specific meteorological environments amplify the harmful impact of O_3_ on cerebrovascular health.

## 4. Discussion

This study provides robust evidence that short-term ambient O_3_ exposure is significantly associated with elevated cerebral infarction risk, with this association substantially modified by meteorological factors. Analysis of five-year hospitalization records from four major Zhengzhou hospitals revealed that heightened stroke risk is correlated not only with increased O_3_ levels, but also with specific meteorological patterns, including the following: moderate temperatures (10–20 °C) on exposure days, low temperatures (<10 °C) at 2–3-day lag, and high temperatures (>30 °C) at 4–6-day lag, forming a distinct bimodal temperature-lag pattern. Additionally, high relative humidity, low wind speed, and low atmospheric pressure were found to collectively strengthen the association between O_3_ exposure and cerebrovascular risk. These findings highlight the complex interactions between air pollution and weather in determining stroke risk, especially among older adults and during the warm season.

During the study period, the ambient O_3_ level in Zhengzhou showed a rising trend. This aligns with national monitoring data from 1341 air quality stations across China, which showed that the annual mean O_3_ concentration increased from 72.4 μg/m^3^ in 2015 to 80.9 μg/m^3^ in 2018 [20].

In addition, the average O_3_ concentration observed in this study reached 109.02 μg/m^3^, which was significantly higher than other cities such as Ganzhou [21] (90.52 μg/m^3^) and Urumqi [22] (67.53 μg/m^3^), as well as the rest of China. These findings suggest that O_3_ pollution in Zhengzhou has become a major environmental health concern. Notably, these findings are specifically tied to Zhengzhou’s urban context and may lack generalizability across regions with distinct climatic conditions. We propose focused validation studies in tropical, arid, and cold climate zones.

Consistent with previous studies, we observed a significant positive association between short-term O_3_ exposure and cerebral infarction hospitalizations. For instance, a time-series study conducted in Shenzhen [9] showed that with a lag time of 0 to 3 days, the risk of hospitalization for cerebral infarction increased by 1.2% for every 10 μg/m^3^ increase in O_3_ concentration (95% CI: 0.2% to 2.2%). Similarly, a French [23] study found that O_3_ exposure was associated with an increased risk of ischemic stroke. Empirical studies from distinct geographical contexts have corroborated O_3_–stroke associations. Research in Corpus Christi [24] demonstrated a significant positive correlation between ambient O_3_ levels and severe stroke incidence, paralleled by German [25] epidemiological findings establishing O_3_ concentration elevation as an independent risk factor for stroke onset. The significant positive associations reported in these studies are generally consistent with those observed in our study. However, studies in London [26] and throughout the United States [27] found no significant correlation between O_3_ exposure and stroke risk. This notable inconsistency may be attributed to the following factors: 1. There are differences in O_3_ background concentrations and pollutant synergies between the regions of the prior study and our current research. In environments with high O_3_ concentrations, human tolerance to pollutant increments may increase, or synergistic effects of other pollutants (such as PM_2.5_ and NO_2_) may exacerbate health risks; 2. There are variations in population susceptibility. Individuals with allergic constitutions or chronic diseases exhibit health effects more rapidly after exposure, whereas healthy populations show longer lag times; 3. Research duration: The length of the study period is a non-negligible factor. Long-term studies can observe lag changes caused by pollutant accumulation effects, while short-term studies may overlook cumulative impacts and only reflect immediate effects.

Cerebral infarction shares multiple pathophysiological pathways with other circulatory system diseases. Previous studies have found that men tend to exhibit greater susceptibility to the cardiovascular effects of air pollutants, although the underlying mechanisms remain uncertain [28,29]. Higher smoking prevalence among men may partially explain their heightened risk, as smoking is a known risk factor for both stroke and pollutant-related vascular injury [30]. However, in our stratified analysis, no statistically significant difference was detected between males and females regarding the effect of O_3_ on cerebral infarction, suggesting that gender may not be a major modifier in this context.

In this study, older adults (>65 years) appeared to have a higher risk of the disease compared to younger adults, which is consistent with the findings of Klompmaker et al. [31] and Lee et al. [32]. However, this contrasts with the findings from Cao Xiuyu et al. [21], who reported that younger people are at higher risk. Such discrepancies may arise from differences in lifestyle, physiological resilience, and exposure behavior across age groups [33]. For example, older adults may have reduced cardiovascular reserve capacity, pre-existing comorbidities, and less ability to adapt to environmental stressors, thereby increasing their vulnerability. These variations highlight the importance of conducting location-specific and population-specific investigations to inform targeted interventions.

While the health effect of O_3_ on circulatory system diseases has been extensively studied [34,35,36], relatively few studies have focused on the interactive effects between O_3_ and meteorological factors on cerebral infarction. Under certain meteorological conditions, O_3_ levels can rise significantly, which may exacerbate their effects on people with underlying medical conditions, such as high blood pressure and diabetes [37]. For instance, high temperatures can increase O_3_ formation, and at the same time, thermal stress may intensify cardiovascular load, thereby elevating stroke risk [38].

Our analysis indicates that co-occurring high ambient O_3_ and adverse meteorological conditions (e.g., elevated humidity, diminished wind speed, reduced atmospheric pressure) demonstrate a significant association with heightened cerebral infarction risk. Notably, a bimodal temporal risk pattern emerged, which is as follows: peak hospitalization risk coincided with moderate temperatures (10–20 °C) on exposure days, while low temperatures (<10 °C) correlated with elevated risk at a 2–3-day lag, and high temperatures (>30 °C) showed a delayed association with risk elevation at a 4–6-day lag. This suggests that temperature influences stroke risk via distinct acute and delayed physiological mechanisms, including autonomic dysfunction, hemodynamic instability, dehydration, inflammation, and coagulation changes. These findings suggest that environmental risk management should incorporate the meteorological context when assessing air pollution-related health threats. The observed interactions imply that O_3_ concentration thresholds for issuing public health warnings may need to be adjusted seasonally or based on concurrent weather conditions. The relevant authorities need to strengthen monitoring of O_3_ concentrations and issue health warnings during high pollution weather conditions, especially for people at high risk of cardiovascular disease.

This study has several strengths. First, the hospitalization data of patients in this study came from four hospitals in different districts of Zhengzhou City, which can better represent the medical level of citizens. Second, it applies robust and widely accepted statistical models (GAM and DLNM) to capture both non-linear and lagged effects, as well as pollutant–meteorological interactions. Nonetheless, several limitations should be acknowledged. First, our exposure assessment was based on ambient monitoring data, which may not reflect individual-level exposure, particularly considering time spent indoors or in microenvironments. Second, limited by the protection of personal privacy information, we do not collect specific information from hospitalized patients, so we cannot adjust for potential confounding factors, such as lifestyle and medication history. Third, the data used are hospital admission data from 2019–2023. During this period, due to the impact of the COVID-19 epidemic, the number of hospitalizations will fluctuate, which may lead to biases in the data. Although we included the entire 5-year period to limit anomalies, future studies should explicitly address the pandemic’s confounding effects. Despite these limitations, our study provides compelling evidence of the adverse effects of O_3_ exposure on cerebral infarction and highlights the amplifying role of specific meteorological conditions. This emphasizes the need for multifactorial environmental health assessments and meteorologically informed air quality management strategies in urban settings.

## 5. Conclusions

This study observed a significant association between short-term O_3_ exposure and elevated risk of hospitalization for cerebral infarction. Notably, this association was modified by the following meteorological conditions: 1. The bimodal temperature effect: The highest risk occurs at moderate temperatures (10–20 °C) on the exposure day; significant risk increases are observed with a 2–3-day lag at low temperatures (<10 °C) and a 4–6-day lag at high temperatures (>30 °C); 2. The synergistic amplification mechanism: High humidity (>80%), low wind speed (<1.5 m/s), and low atmospheric pressure (<1000 hpa) collectively enhance the cerebrovascular hazards of O_3_; 3. Vulnerable populations and seasons: Individuals aged over 80 and the warm season (May–October) exhibit particularly pronounced risks. Thus, this study emphasizes the need to integrate air quality and meteorological indicators to establish a warning system for cerebral infarction, with a focus on protecting elderly populations with pronounced risks.

## Figures and Tables

**Table 1:** Summary statistics of air pollutants, meteorological factors, and cerebral infarction hospitalizations in Zhengzhou, China from 2019 to 2023.

Variables	2019	2020	2021	2022	2023
**Hospitalized cases**					
Total	21.0 (17.0, 27.0)	20.0 (16.0, 25.0)	22.0 (16.5, 28.0)	17.0 (9.5, 22.0)	19.0 (15.0, 25.0)
Gender					
Male	13.0 (9.0, 16.0)	12.0 (9.0, 15.0)	13.0 (10.0, 17.0)	10.0 (6.0, 14.0)	12.0 (9.0, 16.0)
Female	9.0 (6.0, 11.0)	8.0 (6.0, 11.0)	9.0 (6.0, 11.0)	6.0 (3.0, 9.0)	7.0 (5.0, 10.0)
Age (years)					
18–65	9.0 (6.0, 12.0)	9.0 (6.0, 12.0)	10.0 (7.0, 13.0)	7.0 (4.0, 10.0)	8.0 (6.0, 11.0)
65–80	8.0 (5.5, 10.0)	7.0 (5.0, 10.0)	8.0 (5.5, 11.0)	6.0 (3.0, 8.0)	8.0 (5.0, 10.0)
>80	4.0 (3.0, 6.0)	4.0 (2.0, 5.0)	4.0 (2.0, 5.0)	3.0 (1.0, 4.0)	3.0 (2.0, 5.0)
Season ^a^					
Warm	21.0 (17.0, 27.0)	22.0 (18.0, 27.0)	21.0 (16.0, 26.0)	18.5 (9.0, 25.0)	19.0 (16.0, 25.0)
Cold	21.0 (16.0, 27.0)	18.0 (14.0, 23.0)	24.0 (19.0, 28.0)	15.0 (10.0, 20.0)	19.0 (14.0, 24.0)
**Air pollutants ^b^**					
O_3_ (μg/m^3^)	104.1 (68.3, 150.2)	102.1 (70.7, 149.9)	95.0 (65.0, 138.0)	106.0 (63.0, 157.5)	106.0 (64.75, 145.0)
NO_2_ (μg/m^3^)	41.0 (31.0, 54.0)	35.0 (25.7, 48.0)	29.0 (20.0, 41.0)	24.6 (17.7, 33.6)	26.0 (18.0, 37.0)
CO (mg/m^3^)	0.8 (0.7, 1.1)	0.7 (0.6, 0.9)	0.7 (0.6, 0.8)	0.6 (0.5, 0.8)	0.6 (0.5, 0.7)
SO_2_ (μg/m^3^)	9.0 (6.0, 12.0)	8.0 (5.0, 11.0)	8.0 (5.0, 10.0)	7.6 (5.7, 9.4)	6.3 (4.6, 8.5)
PM_10_ (μg/m^3^)	91.0 (67.5, 130.0)	81.0 (58.0, 108.0)	73.0 (50.0, 108.0)	70.5 (51.1, 102.4)	65.1 (44.5, 106.9)
PM_2.5_ (μg/m^3^)	39.0 (28.0, 69.5)	37.0 (25.7, 60.0)	32.0 (22.0, 54.0)	32.7 (22.3, 55.1)	31.3 (21.0, 51.8)
**Meteorological factors**					
Temperature (°C)					
Warm	26.1 (21.7, 28.7)	25.7 (22.1, 27.8)	25.8 (21.2, 28.5)	17.1 (12.4, 28.3)	26.2 (20.0, 29.2)
Cold	7.2 (3.0, 13.5)	7.2 (3.5, 14.1)	9.3 (5.5, 13.5)	11.3 (3.0, 16.7)	7.1 (3.3, 13.4)
Relative Humidity (%)					
Warm	58.0 (45.2, 69.0)	69.0 (50.0, 81.0)	74.0 (57.5, 87.0)	62.0 (46.0, 74.0)	66.0 (54.0, 74.0)
Cold	55.0 (42.0, 69.5)	57.5 (42.0, 74.2)	50.0 (32.0, 69.0)	57.0 (43.0, 73.5)	53.0 (38.5, 66.0)
Wind speed (m/s)					
Warm	1.7 (1.4, 2.1)	1.7 (1.3, 2.1)	1.8 (1.3, 2.2)	1.7 (1.3, 2.2)	1.3 (1.0, 1.9)
Cold	1.5 (1.2, 2.1)	1.6 (1.2, 2.2)	1.8 (1.3, 2.5)	1.5 (1.1, 1.9)	1.6 (1.2, 2.3)
Air pressure (hpa)					
Warm	995 (991, 998)	994 (991, 1001)	994 (991, 1000)	1005 (994, 1011)	996 (991, 1002)
Cold	1010 (1004, 1015)	1011 (1006, 1015)	1009 (999, 1013)	1009 (1002, 1013)	1010 (1003, 1016)

Note: ^a^ warm season: May to October; cold season: November to April. ^b^ O_3_: maximum daily 8-h average; other pollutants: 24-h average.

**Table 2:** Spearman’s correlation coefficients between air pollutants and meteorological.

	O_3_	NO_2_	CO	SO_2_	PM_10_	PM_2.5_	T	RH	WD	AP
O_3_	1.00									
NO_2_	−0.32 **	1.00								
CO	−0.37 **	0.58**	1.00							
SO_2_	−0.26 **	0.67 **	0.40 **	1.00						
PM_10_	−0.18 **	0.45 **	0.38 **	0.43 **	1.00					
PM_2.5_	−0.38 **	0.61 **	0.80 **	0.49 **	0.66 **	1.00				
T	0.73 **	−0.37 **	−0.42 **	−0.44 **	−0.23 **	−0.50 **	1.00			
RH	−0.15 **	−0.08 **	0.33 **	−0.44 **	−0.21 **	0.13 **	0.046	1.00		
WD	0.04 *	−0.35 **	−0.25 **	−0.10 **	−0.02	−0.20 **	0.09 **	−0.02 **	1.00	
AP	−0.64 **	0.33 **	0.31 **	0.41 **	0.15 **	0.37 **	−0.89 **	−0.10 **	−0.12 **	1.00

Abbreviations: T, temperature; RH, relative humidity; WD, wind speed; AP, air pressure; PM_2.5_, fine particulate matter; PM_10_, inhalable particulate matter; SO_2_, sulfur dioxide; NO_2_, nitrogen dioxide; CO, carbon monoxide; O_3_, ozone. * *p* < 0.05; ** *p* < 0.01.

**Table 3:** Single-day lag effects of ozone on hospitalization risk for cerebral infarction (ER, 95% CI).

Lag Day		Gender (%)		Age (%)			Season (%)	
	Total	Male	Female	18–65	66–80	>80	Warm	Cold
0	**0.11 (0.08, 0.14)**	**0.12 (0.07, 0.16)**	**0.12 (0.06, 0.16)**	**0.12 (0.07, 0.16)**	**0.08 (0.03, 0.13)**	**0.13 (0.06, 0.20)**	**0.08 (0.03, 0.12)**	0.01 (−0.06, 0.05)
1	**0.08 (0.05, 0.10)**	**0.09 (0.03, 0.14)**	**0.10 (0.05, 0.14)**	**0.07 (0.03, 0.12)**	**0.08 (0.03, 0.12)**	**0.07 (0.01, 0.14)**	0.03 (0.00, 0.06)	0.01 (−0.04, 0.06)
2	**0.08 (0.05, 0.11)**	**0.07 (0.04, 0.11)**	**0.10 (0.05, 0.14)**	**0.08 (0.04, 0.12)**	**0.10 (0.06, 0.15)**	**0.06 (0.00, 0.13)**	0.02 (0.00, 0.05)	0.03 (−0.01, 0.09)
3	**0.07 (0.04, 0.10)**	**0.06 (0.03, 0.10)**	**0.08 (0.04, 0.13)**	0.06 (0.02, 0.10)	**0.09 (0.04, 0.13)**	**0.07 (0.01, 0.13)**	−0.01(−0.04, 0.01)	**0.06 (0.02, 0.12**)
4	**0.08 (0.05, 0.10)**	**0.09 (0.05, 0.12)**	0.06 (0.01, 0.10)	**0.08 (0.04, 0.12)**	**0.09 (0.04, 0.13)**	0.04 (0.00, 0.10)	0.01 (−0.03, 0.04)	0.03 (−0.01, 0.08)
5	**0.06 (0.03, 0.09)**	**0.06 (0.02, 0.09)**	**0.06 (0.01, 0.10)**	**0.06 (0.02, 0.10)**	**0.05 (0.01, 0.10)**	**0.09 (0.02, 0.15)**	−0.01 (−0.04, 0.02)	0.01 (−0,04, 0.03)
6	**0.05 (0.02, 0.08)**	0.05 (0.02, 0.09)	0.05 (0.01, 0.10)	0.04 (0.01, 0.02)	**0.07 (0.03, 0.12)**	0.06 (0.00, 0.12)	−0.01 (−0.04, 0.03)	−0.01 (−0.07, 0.03)
7	**0.09 (0.06, 0.11)**	**0.09 (0.06, 0.13)**	**0.07 (0.03, 0.12)**	**0.07 (0.03, 0.11)**	**0.11 (0.07, 0.15)**	**0.07 (0.01, 0.13)**	0.03 (0.01, 0.06)	−0.01 (−0.06, 0.04)

Note: The statistically significant estimates are bolded (*p* < 0.05).

**Table 4:** Cumulative lag effects of ozone on hospitalization risk for cerebral infarction (ER, 95% CI).

Lag Day		Gender (%)		Age (%)			Season (%)	
	Total	Male	Female	18–65	66–80	>80	Warm	Cold
0–1	**0.13 (0.10, 0.16)**	**0.11 (0.07, 0.15)**	**0.11 (0.07, 0.16)**	**0.12 (0.08, 0.17)**	**0.08 (0.03, 0.13)**	**0.16 (0.08, 0.23)**	**0.08 (0.05, 0.12)**	−0.01 (−0.06, 0.05)
0–2	**0.11 (0.08, 0.14)**	**0.09 (0.03, 0.15)**	**0.10 (0.05, 0.14)**	**0.07 (0.03, 0.12)**	**0.07 (0.03, 0.12)**	**0.13 (0.06, 0.20)**	0.02 (−0.05, 0.06)	0.00 (−0.04, 0.06)
0–3	**0.07 (0.05, 0.10)**	**0.07 (0.03, 0.11)**	**0.09 (0.05, 0.14)**	**0.08 (0.04, 0.12)**	**0.10 (0.05, 0.14)**	**0.07 (0.00, 0.14)**	0.01 (−0.01, 0.05)	0.03 (−0.01, 0.09)
0–4	**0.08 (0.05, 0.11)**	**0.06 (0.02, 0.09)**	**0.08 (0.03, 0.12)**	**0.06 (0.02, 0.10)**	**0.08 (0.04, 0.13)**	**0.06 (0.00, 0.12)**	−0.01 (−0.05, 0.02)	**0.06 (0.02, 0.12)**
0–5	**0.07 (0.04, 0.10)**	0.08 (0.05, 0.12)	0.05 (0.01, 0.09)	**0.08 (0.04, 0.12)**	**0.08 (0.03, 0.12)**	0.06 (0.00, 0.12)	0.01 (−0.04, 0.04)	0.02 (−0.02, 0.08)
0–6	**0.05 (0.03, 0.08)**	**0.05 (0.02, 0.08)**	**0.05 (0.01, 0.09)**	**0.05 (0.01, 0.09)**	**0.05 (0.00, 0.09)**	**0.03 (0.00, 0.10)**	−0.01 (−0.04, 0.02)	0.00 (−0,04, 0.05)
0–7	**0.05 (0.02, 0.07)**	**0.05 (0.01, 0.08)**	0.04 (0.00, 0.09)	0.03 (0.00, 0.07)	0.07 (0.02, 0.11)	**0.08 (0.02, 0.14)**	−0.01 (−0.04, 0.01)	−0.02 (−0.07, 0.03)

Note: The statistically significant estimates are bolded (*p* < 0.05).

## Data Availability

Due to privacy protection restrictions, the data generated by this study are not yet made public. However, under the condition of providing a reasonable research plan and obtaining ethical approval, the relevant data can be requested for access by contacting the corresponding author.
